# Evaluating Differences in Aluminum Exposure through Parenteral Nutrition in Neonatal Morbidities

**DOI:** 10.3390/nu9111249

**Published:** 2017-11-16

**Authors:** Megan Fortenberry, Lela Hernandez, Jacob Morton

**Affiliations:** 1 University of North Carolina Health Care, Chapel Hill, NC 27514, USA; megan.fortenberry@unchealth.unc.edu; 2 Wesley Children’s Hospital, 550 N Hillside, Wichita, KS 67214, USA; 3 Saint Vincent Hospital, 123 Summer St, Worcester, MA 01608, USA; Jacob.morton@stvincenthospital.com

**Keywords:** parenteral nutrition, aluminum, neonate

## Abstract

Aluminum is a common contaminant in many components of parenteral nutrition, especially calcium and phosphate additives. Although long-term effects have been described in the literature, short-term effects are not well-known. Currently, the Food and Drug Administration recommends maintaining aluminum at <5 mcg/kg/day. This was a single center, retrospective case-control study of 102 neonatal intensive care unit patients. Patients were included if they had a diagnosis of necrotizing enterocolitis, rickets/osteopenia, or seizures and received at least 14 days of parenteral nutrition. Patients were matched 1:1 with control patients by gestational age and birth weight. Mean total aluminum exposure for the 14 days of parenteral nutrition was calculated using manufacturer label information. Differences in mean aluminum exposure between cases and controls, as well as subgroup analysis in those with renal impairment or cholestasis, was conducted. Aluminum exposure in patients meeting inclusion criteria closely mirrored the aluminum exposure of control patients. The difference in aluminum exposure was not found to be statistically significant, except in patients with cholestasis. Although the study found no difference in aluminum exposure in short-term complications with neonates, long-term complications are well established and may warrant the need to monitor and limit neonatal aluminum exposure.

## 1. Introduction

Aluminum serves no known biological role in the human body. Humans are naturally exposed to aluminum through drinking water, foods, medications, dust, and deodorant [1]. Additionally, aluminum is a common contaminant in many components of parenteral nutrition, especially calcium and phosphate additives. Under normal circumstances, the human body has natural defense mechanisms that prevent significant absorption of ingested aluminum. An intact gastrointestinal tract typically allows less than 1% absorption of aluminum [1]. Of the aluminum that enters the bloodstream, 99% is excreted through the kidneys.

Preterm infants require high amounts of calcium and phosphate for bone mineralization. This, coupled with poor renal function, predispose preterm infants to a high risk for aluminum toxicity when fed parenterally. Reports of aluminum toxicity have been described since the 1970s [2]. Potential long-term effects of aluminum include neurotoxicity, anemia, bone disease, and cholestasis [3,4,5,6,7,8,9,10,11,12]. As such, the Food and Drug Administration recommends maintaining aluminum exposure at less than 5 mcg/kg/day [13].

Currently, there is little research examining the effects of parenteral aluminum exposure on neonatal morbidities in an acute inpatient setting. The objective of this study was to determine if estimated mean cumulative aluminum exposure as part of parenteral nutrition is increased in neonates with poor outcomes, including necrotizing enterocolitis, rickets/osteopenia, and/or seizures when compared with control patients. Diagnoses were selected based on previous literature establishing a relationship between aluminum exposure and development of neurotoxicity and inhibition of proper bone maturation [3,4,5,6,7,8,9,10,11,12,14]. Necrotizing enterocolitis was selected as an inclusion diagnosis based on the direct relationship of the disease state with extended parenteral nutrition requirements. Secondary outcomes included: evaluation of mean cumulative aluminum exposure in neonates with renal dysfunction or cholestasis as well as determination of the doses of aluminum to which more cases were exposed, if a significant difference existed between cases and controls.

## 2. Materials and Methods

### 2.1. Study Design

This was a single center, retrospective, case-control study. All data was collected utilizing electronic medical records. Data collected included: gestational age, birth weight, daily weight, diagnoses, and parenteral nutrition (PN) formulas. Parenteral nutritional formulas, as well as daily weight, were entered into an aluminum calculator developed by investigators in Microsoft Excel^©^ (Microsoft Professional Office 2016; Microsoft Corporation, Redmond, WA, USA) utilizing current manufacturer labeling to determine daily exposure to aluminum in mcg/kg/day (Table 1).

Each PN formula was entered into the calculator by the primary investigator. Daily aluminum exposure over the first 14 days in each patient were added to determine the cumulative aluminum exposure in mcg/kg. Data was maintained utilizing a de-identified list. This study was approved by the Wichita Medical Research and Education Foundation Institutional Review Board.

### 2.2. Patient Selection

Patients were included in the study sample if they received at least 14 days of parenteral nutrition between 1 June 2013 and 30 June 2016. All patients who received 14 days of parenteral nutrition during the preselected timeframe were retrieved from the electronic database. From there, cases were selected if they had a diagnosis consistent with necrotizing enterocolitis, rickets/osteopenia, or seizures based on diagnoses in Neodata^®^ (Isoprime, Lisle, IL, USA). Control patients were selected if they had received at least 14 days of parenteral nutrition without development of any of the inclusion diagnoses. Controls were selected using a random sequence generator. Cases were matched 1:1 to a control patient based on gestational age and birth weight. Patients were excluded if they were diagnosed with any inclusion diagnoses prior to the initiation of parenteral nutrition, if they received less than 14 days of parenteral nutrition, or if they were small for gestational age. There were no gestational age or birth weight requirements for inclusion in the study.

### 2.3. Outcomes

The primary outcome of this study was to determine if estimated mean cumulative aluminum exposure as part of parenteral nutrition is increased in neonates with poor outcomes, including necrotizing enterocolitis, rickets/osteopenia, and/or seizures. Secondarily, the study aimed to evaluate the mean cumulative aluminum exposure in neonates with renal dysfunction, defined as serum creatinine >1.5 mg/dL, or cholestasis, defined as direct bilirubin >2 mg/dL. An additional secondary outcome included determining the doses of aluminum (mcg/kg/day) to which more cases were exposed, if a significant difference in exposure existed between cases and controls.

### 2.4. Statistical Analysis

Based on an alpha of 5% and an 80% power to detect a difference in risk of development of inclusion diagnoses between cases and control of approximately 2.5 (i.e., odds ratio = 2.5), we calculated that 85 cases and 85 controls need to be included [14,15]. Differences in mean aluminum exposure between cases and controls were analyzed using a Student’s *t*-test. If a statistically significant difference (*p* < 0.05) in aluminum exposure was found between cases and controls, a classification and regression tree (CART) analysis would be conducted to determine the doses of aluminum for which more cases were exposed.

## 3. Results

A total of 156 patients were selected between 1 June 2013 and 30 June 2016. Of these, 54 patients were excluded due to receiving less than 14 days of parenteral nutrition. The remaining 102 resulted in 51 matched pairs. There was an even split of male and female patients with 51 of each. Patients were on average 27 weeks and 3 days gestational age (24 weeks, 2 days–33 weeks, 6 days) and weighed 1.029 kg (0.59 kg–2.223 kg) at birth. Thirty-one patients had a diagnosis of seizures, nineteen patients had a diagnosis of necrotizing enterocolitis, and twenty-three patients had osteopenia/rickets. Of the patients included, twenty had at least two inclusion diagnoses and two patients met all three inclusion diagnoses. Regarding secondary endpoints, ten patients met inclusion criteria for renal dysfunction and three patients met criteria for cholestasis. Demographic information can be found in Table 2.

Total aluminum exposure in all patients meeting inclusion criteria was 78.8 mcg/kg/day when compared with control patients of 79.2 mcg/kg/day (*p* = 0.87). Patients with a diagnosis of seizures had a total aluminum exposure of 78.3 mcg/kg/day when compared with control patients of 78.7 mcg/kg/day (*p* = 0.91). Regarding necrotizing enterocolitis, patients with the diagnosis had a 79.1 mcg/kg/day while matched control patients had a total exposure of 80.5 mcg/kg/day (*p* = 0.72). Total aluminum exposure in patients with osteopenia or rickets was 80.8 mcg/kg/day when compared with controlled patients whose aluminum exposure was 79.6 mcg/kg/day (*p* = 0.76). Twenty patients had multiple inclusion diagnoses. The total aluminum exposure of case patients who had multiple inclusion diagnoses was 80.1 mcg/kg/day versus average 78.6 mcg/kg/day (*p* = 0.75).

A total of 10 patients met the inclusion criteria for renal dysfunction. The average aluminum exposure of these 10 patients was 80.4 mcg/kg/day when compared with 78.9 mcg/kg/day in the 92 patients who did not have renal dysfunction (*p* = 0.7). Three patients met the inclusion criteria for cholestasis. The average aluminum exposure in patients with cholestasis was 91.7 mcg/kg/day compared with 78.7 mcg/kg/day in the 99 patients who did not meet inclusion criteria for cholestasis (*p* = 0.04).

## 4. Discussion

The utilization of parenteral nutrition (PN) is a routine practice in the neonatal intensive care unit. Aluminum is commonly found as a contaminant in many components of parenteral nutrition, especially calcium and phosphate additives. A study conducted by Moreno and colleagues, found that parenteral nutrition solutions were the main source of aluminum exposure in neonates, representing 88.7% of aluminum intake [16]. Reports of aluminum toxicity first appeared in the 1970’s [3]. However, to our knowledge this is the first study to assessing the relationship between aluminum exposure through parenteral nutrition and acute morbidities in neonatal patients.

An influential study published by Bishop and colleagues examined 227 infants in the neonatal intensive care unit. The infants were randomized to receive either standard aluminum or aluminum-depleted intravenous-feeding solutions. In infants who received more than 10 days of parenteral nutrition, the Mental Development Index of those receiving the standard aluminum PN was found to be statistically lower than those who received the aluminum-depleted intravenous-feeding solutions (*p* = 0.02). In addition, infants who received the standard aluminum intravenous-feeding solutions were statistically more likely to have a Mental Development Index below 85 points, which could increase their risk of educational impairment (*p* = 0.03). It was estimated that infants would lose around one Mental Development Index point for each day on the standard aluminum solution [4]. However, it is important to note that infants with a diagnosis of neuromotor impairment were excluded as this impairment could render the assessment of the Bayley Mental Scale inaccurate.

A follow-up study by Fewtrell and colleagues evaluated what effect the standard aluminum and aluminum-depleted intravenous-feeding solutions had on the bone development of infants from the Bishop study. Adolescents who had participated in the Bishop study as infants were invited back for assessment of bone area, bone mineral content, and bone mineral density. Patients who had a high exposure to aluminum (55 mcg/kg/day) had significantly lower hip bone mineral content after adjusting for confounding variables (*p* = 0.02) [12].

In addition, the rate of parenteral nutrition-associated cholestasis ranges from 7.4% to 84% [2]. Previous research has shown that hepatic aluminum concentrations were between 5 and 27 times higher than normal concentrations in infants receiving parenteral nutrition [17]. Further studies in animals found that animals who were exposed to parenteral nutrition had significant increases in both the serum bile acids and alkaline phosphatase levels when compared with animals who had not received parenteral nutrition [18,19]. Rats who had received parenteral nutrition for 14 days had higher concentrations of serum bile acids than those who had received parenteral nutrition for 7 days [19]. Of the rats who received the parenteral nutrition for 14 days, the group that received high doses of aluminum (5 mg/kg/day) had a 33% reduction in biliary flow [19].

A previous study demonstrated that approximately 90% of pediatric patients between the ages of 6 months and 17 years receiving parenteral nutrition had plasma aluminum concentrations above the reference range of 0–371 mmol/L [20]. Of these patients, approximately 20% of patients had an aluminum concentration 5–8 times the upper limit of normal [20]. Due to the retrospective nature of this study, aluminum exposure was calculated versus drawn serum levels. A previous study showed that there is no difference in aluminum serum concentrations with various levels of aluminum exposure [21]. However, at our institution serum aluminum levels are not routinely drawn, limiting our ability to evaluate true total aluminum exposure.

For consistency within our study, the aluminum content listed by the manufacturer was used to build the aluminum calculator. However, in 2012 Poole and colleagues demonstrated that components of parenteral nutrition may contain less aluminum than labeled by the manufacturer [22]. Despite finding that parenteral nutrition components contained less aluminum than the label indicated, Poole and colleagues identified that neonatal compounded parenteral nutrition still contained 3–5 times more than the United States Food and Drug Administration (FDA) recommended “safe limit.” The study concluded that even when making a conscious effort to use the least contaminated parenteral nutrition products, daily aluminum exposure still totaled to 8.8–12.9 mcg/kg/day [22]. Notably, the highest measured and calculated aluminum content was found among the smallest patients.

Despite literature describing the complications associated with aluminum exposure, our study supports previous claims of the inability to provide nutrition and maintain aluminum exposure less than 5 mcg/kg/day. In fact, of the 1428 parenteral nutrition formulas analyzed during the study period, only seven formulas contained aluminum in amounts below the FDA “safe limit.” However, in spite of providing aluminum beyond the FDA limit in the majority of our patients, mean cumulative aluminum exposures were similar in those with and without the outcomes of interest. There were a few limitations with our study. In some instances, the neonates involved in our study did not receive all 14 of their PN days consecutively. In addition, only the first 14 days of parenteral nutrition was evaluated, so it is possible the total cumulative aluminum exposure may differ between patients with diagnoses versus control patients if patients with inclusion diagnoses had received more total days of parenteral nutrition. Other limitations of this study include that patients were only matched on weight and gestational age, which may not account for all compounding factors. Specific amounts of calcium and phosphate were not evaluated in related to osteopenia/rickets diagnoses. Finally, the study fell short of enrolling enough patients to meet predefined power.

## 5. Conclusions

In conclusion, mean cumulative aluminum exposure in neonates receiving at least 14 days of parenteral nutrition was similar among those with and without necrotizing enterocolitis, seizures, and/or osteopenia/rickets. The majority of patients were exposed to aluminum concentrations above the current FDA limit. However, larger, high-quality studies are needed to further assess the relationship between aluminum exposure and morbidity.

## Figures and Tables

**Table 1:** Manufacturer Aluminum Content.

Product	Brand	Aluminum Content (mcg/L)
Dextrose	Hospira	25
Amino Acids	Hospira	25
Sodium Chloride	APP/Fresenius ^1^	200
Sodium Acetate	APP/Fresenius	400
Sodium Phosphate	APP/Fresenius	16,000
Potassium Chloride	APP/Fresenius or Hospira	100
Potassium Acetate	Exela or Hospira	200
Potassium Phosphate	APP/Fresenius	32,800
Calcium Gluconate	American Regent	12,500
Magnesium Sulfate	American Regent	12,500
Fat Emulsion	APP/Fresenius	25

^1^ APP-American Pharmaceutical Partners.

**Table 2:** Patient Demographic Data.

	Case Patients (*n* = 51)	Control Patients (*n* = 51)
Average Gestational Age	27 weeks, 2 days (±1 week, 3 days)	27 weeks, 3 days (±1 week, 3 days)
Average Birth Weight	1.029 kg (±0.32 kg)	1.029 kg (±0.32 kg)
Gender, male	27 (53%)	24 (47%)
Seizures	31 diagnoses (61%)	-
Necrotizing Enterocolitis	19 diagnoses (19%)	-
Osteopenia/Rickets	23 diagnoses (45%	-
